# Genome scan for body mass index and height in the Framingham Heart Study

**DOI:** 10.1186/1471-2156-4-S1-S91

**Published:** 2003-12-31

**Authors:** Frank Geller, Astrid Dempfle, Tilman Görg

**Affiliations:** 1 Institute of Medical Biometry and Epidemiology, Philipps-University of Marburg, Bunsenstrasse 3, Marburg, 35037 Germany

## Abstract

**Background:**

Body mass index (BMI) and adult height are moderately and highly heritable traits, respectively. To investigate the genetic background of these quantitative phenotypes, we performed a linkage genome scan in the extended pedigrees of the Framingham Heart Study. Two variance-components approaches (SOLAR and MERLIN-VC) and one regression method (MERLIN-REGRESS) were applied to the data.

**Results:**

Evidence for linkage to BMI was found on chromosomes 16 and 6 with maximum LOD scores of 3.2 and 2.7, respectively. For height, all markers showing a LOD score greater than 1 in our analysis correspond to previously reported linkage regions, including chromosome 6q with a maximum LOD score of 2.45 and chromosomes 9, 12, 14, 18, and 22. Regarding the analysis, the three applied methods gave very similar results in this unselected sample with approximately normally distributed traits.

**Conclusion:**

Our analysis resulted in the successful identification of linked regions. In particular, we consider the regions on chromosomes 6 and 16 for BMI and the regions on chromosomes 6, 9, and 12 for stature interesting for fine mapping and candidate gene studies.

## Background

In recent years, about 20 genome scans for obesity and obesity-related phenotypes have been published. Many of these focussed on obesity using the affected sib-pair design, which offers good power compared with the necessary recruitment effort. On the other hand, many epidemiological studies or genome scans for common diseases come up with large and well characterized samples. If a sufficient number of the recruited individuals is related and DNA or genotype information is also available, linkage analysis for several traits can be conducted. The Genetic Analysis Workshop 13 provides genetic and anthropometrical data from 330 general pedigrees of the Framingham Heart Study. Thus, we studied the genetics of body mass index (BMI) and height using a two-stage approach, which ensures that all individuals can be analyzed together. First, we built regression models for the phenotypes to obtain a single adjusted trait value for each individual. At the second stage we performed linkage analyses incorporating all genotyped individuals.

## Methods

### Study group

The individuals from the Framingham Heart Study were recruited at two time points (the original cohort in 1948 and the offspring cohort in 1971) from the general population excluding those with cardiovascular diseases, heart attack, or stroke. Almost all participants were of Caucasian origin. From the 330 largest pedigrees with 4692 members, DNA was available for 1702 individuals, who were genotyped for 401 markers on the 22 autosomes. The positions of the markers were from the Marshfield website: . We used the sex-averaged positions converted to the Haldane mapping function. Phenotypic information is provided for 2885 persons (1213 from the original cohort). Detailed information about the Framingham Heart Study is given at .

### Condensing and trimming of pedigrees

We condensed and trimmed the given pedigrees to enable effective multi-point linkage analysis with MERLIN [1]. Condensation was done without losing linkage information because only untyped individuals were discarded. Here, ungenotyped persons without children and untyped founders with only one child were removed, since they are not informative for linkage. After this step, four families were removed because they had no informative relationship left and four families fell into two unrelated branches. Finally, 14 families, which were still too large to allow some of the planned analyses, were trimmed by breaking some relationships that carried the least linkage information. This resulted in a total of 346 pedigrees with 2656 individuals used in all analyses. The pedigree size ranged from 4 to 18 individuals in two to four generations.

### Phenotype definition

The available longitudinal phenotypic information for each person was transformed into one specific value for each trait. For BMI, we defined an individual mean that accounts for all available BMI measures. This allowed us to circumvent the problem of missing values at single time points. The phenotype height was investigated as the maximum of the height measurements. Regression models for BMI and height were built for each sex in the original and the offspring cohort separately.

BMI was log-transformed to account for the underlying skewed distribution and adjusted for age and smoking (cigarettes per day). To get an estimate of an overall mean for a person which accounts for the multiple measures, all available examinations of each person between the age of 20 and 70 years were considered and a class variable for each individual was incorporated. Thus, the observation for the i^th ^individual at time t is modelled as:

log (*BMI*)_*it *_= μ + π_*i *_+ β_1 _*a*_*it *_+ β_2 _*c*_*it *_+ *e*_*it*_,

with μ as the overall mean, π_i _as the individual effect, a_it _as the age at time t (including quadratic and higher order terms depending on the sex-specific and cohort-specific model), c_it _as the cigarette consumption at time t, and e_it _as the residual at time t. This model gives one value for the least squares mean π_i _for each individual. The standardized values of π_i _are approximately normally distributed and were used as phenotypic information in all linkage analyses.

For height, the maximum height of each individual older than 18 years was modelled, adjusted for age at first examination to account for the different years of birth. The model for the i^th ^individual is:

max (*height*)_*i *_= μ + β *a*_*i *_+ *e*_*i*_

with μ as the overall mean, a_i _as the age at first examination, and e_i _as the residual. The standardized residuals are approximately normally distributed and were taken as height variables in the linkage analyses.

### Linkage analysis methods

Multipoint linkage analyses for both the longitudinal BMI and the height phenotype were done with the variance-components (VC) models implemented in MERLIN [1] and SOLAR [2] as well as with the model-free regression method MERLIN-REGRESS [3]. VC methods model the phenotypic variance that is explained by the estimated identity-by-descent (IBD) sharing at a chromosomal position. The idea behind MERLIN-REGRESS is to regress the estimated IBD sharing between relative pairs on the squared sums and squared differences of their trait values. MERLIN (REGRESS and VC) calculates exact IBD sharing probabilities using the Lander-Green algorithm with sparse gene flow trees and can handle pedigrees up to about 20 individuals for multi-point analysis [1,3]. On the other hand, SOLAR estimates multi-point IBD sharing probabilities with a generalization of the Fulker method [2] and has no restriction on the pedigree size.

## Results

**Figure 1:**
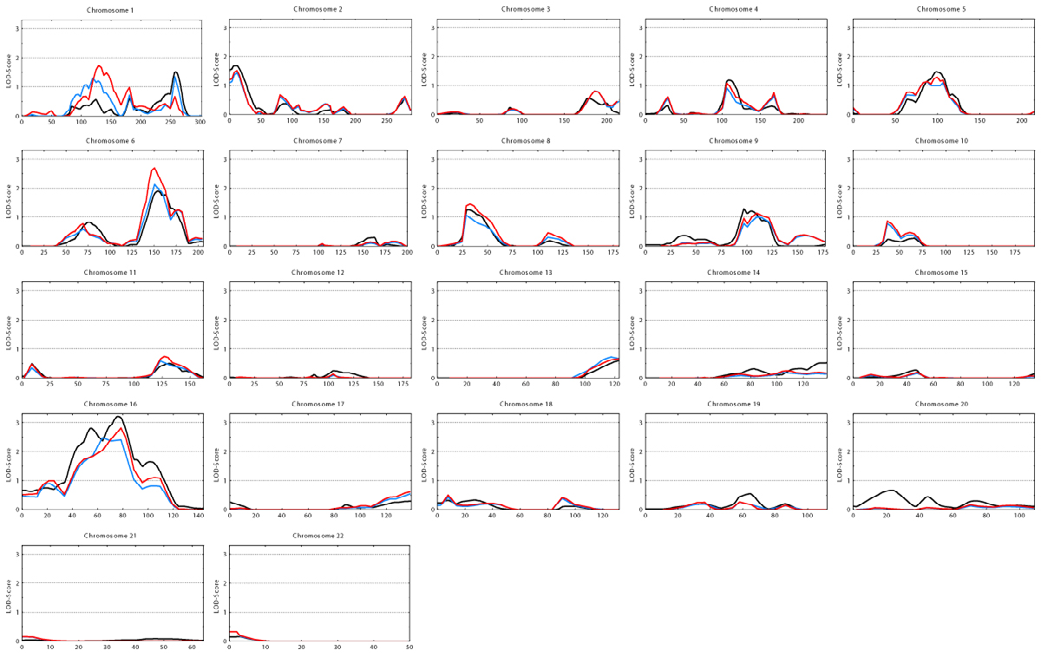
**Multi-point LOD scores for BMI. **Analysis method: SOLAR (black line), MERLIN-VC (red line), MERLIN-REGRESS (blue line). For ease of presentation, negative LOD scores from MERLIN-REGRESS were set to 0. Positions are given in cM.

**Figure 2:**
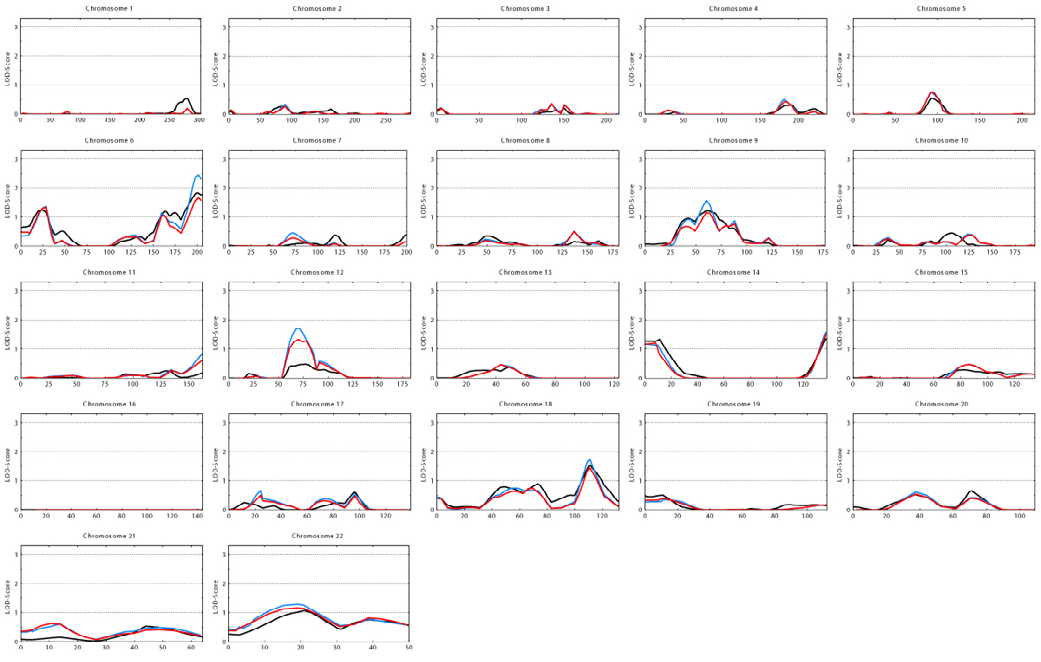
**Multi-point LOD scores for height. **Analysis method: SOLAR (black line), MERLIN-VC (red line), MERLIN-REGRESS (blue line). For ease of presentation, negative LOD scores from MERLIN-REGRESS were set to 0. Positions are given in cM.

Figures 1 and 2 show multi-point LOD score results for BMI and height from SOLAR, MERLIN-VC, and MERLIN-REGRESS for the 22 autosomes. Tables 1 and 2 give all LOD scores greater than 1. The heritabilities of the longitudinal adult BMI and maximum height were estimated by the VC methods as 0.45 and 0.8, respectively.

### BMI

LOD scores greater than 1 were observed on chromosomes 1, 2, 4, 5, 6, 8, 9, and 16. The maximum LOD scores for BMI were found on chromosome 16 with 3.21 for SOLAR, 2.81 for MERLIN-VC, and 2.47 for MERLIN-REGRESS with a 1-LOD support interval reaching from 45 to 85 cM. A second interesting region was identified on chromosome 6 with LOD scores of 1.90 to 2.70 depending on the analysis method.

### Height

For chromosomes 6p, 6q, 9, 12, 14, 18, and 22, LOD scores greater than 1 were obtained with at least one analysis method. The strongest evidence for linkage to height was found near the q-ter of chromosome 6, with a LOD score of 2.45 for MERLIN-REGRESS and a 1-LOD support interval spanning from 190 to 204 cM. The VC methods gave LOD scores of 1.83 and 1.67 at the same position.

### Comparison of methods

For this unselected sample both the VC methods and the regression method implemented in MERLIN are valid and showed remarkably close agreement over the whole genome (see Figs. 1 and 2). The two MERLIN variants (VC and REGRESS) with different statistical methods showed more similarity in the general shape and level of the LOD score curve than did the two different implementations of VC methodology (MERLIN-VC and SOLAR). This might have been due to the different IBD estimation procedures implemented, which seemed to have more influence than the statistical methods. With all three methods, the four highest LOD scores for BMI occurred at the same positions and were of comparable magnitude. For height there was also complete agreement at the highest peak on chromosome 6, and close agreement for the peaks on chromosomes 18 and 14. Among all 18 regions with a LOD score above 1 with any method, there were only four where one of the methods produced a LOD smaller than 1.

## Discussion

We performed a linkage genome scan for the quantitative phenotypes BMI and adult height in the extended pedigrees of the Framingham Heart Study. Analyses were conducted using two VC approaches (SOLAR and MERLIN-VC) and one regression method (MERLIN-REGRESS). These linkage analysis methods were applied to the same data, thus allowing us to compare the results. All three methods can be used for this unselected sample with approximately normally distributed traits. The observed results are remarkably similar over the whole genome and show close agreement in the positions and magnitudes of the highest LOD scores. We cannot assess which method has the most power, since no functional relationships to BMI or height are proven for genes in the regions with high LOD scores, and therefore we cannot recommend a specific method from this application to real data. Using simulated data of normally distributed traits, Sham et al. [3] showed that MERLIN-REGRESS and MERLIN-VC give similar results in small sibships and that MERLIN-REGRESS has more power in larger sibships (6 sibs). In our application there was no obvious difference between MERLIN-REGRESS and MERLIN-VC in these medium sized pedigrees (average 7.7 individuals).

Considering the numerous studies for BMI and BMI-related phenotypes, annually reviewed in the human obesity gene map [4], we concentrated on our two regions on chromosomes 6 and 16 and compared them with already published linkage findings. Wu et al. [5] analyzed eight individual studies conducted in the context of blood pressure for BMI. Their GENOA study group of White Americans displayed a LOD score of 2.55 in a region reaching from 66 to 88 cM on chromosome 16 corresponding with our peak. However, this result of Wu et al. [5] was not supported by the other two samples of White Americans in their study. For the region on chromosome 6q, Feitosa et al. [6] report a LOD score of 1.6 for BMI in two combined US samples. Arya et al. [7] found significant linkage between a factor consisting of BMI, leptin, and fasting-specific insulin and the region ranging from D6S403 (142 cM, LOD = 4.2) to D6S264 (179 cM, LOD = 4.9) in nondiabetics from Mexican American families, while for the BMI-related phenotype fasting glucose and specific insulin, Duggirala et al. [8] obtained a LOD score of 4.1 near the marker D6S403 in these families.

For the phenotype adult height, we were able to identify several regions that showed evidence for linkage in some of the six genome scans published to date. In particular on chromosome 6q we had a broad peak with a maximum LOD of 2.45 at 201 cM. Interestingly, Hirschhorn et al. [9] and Xu et al. [10], reported LODs of 3.85 at 159 cM and 3.06 at 155 cM, respectively. In this region we obtained a LOD score of 1.19 and it remains unclear if the maximum LOD scores on 6q result from the same locus. Substantial corroborative evidence exists also from Hirschhorn et al. [9], Xu et al. [10], and Perola et al. [11] for the regions on chromosomes 6p, 9, 12, 14, 18, and 22 (see Table 3). However, there was no overlap with the putative linkage regions reported by Thompson et al. [12] and Wiltshire et al. [13]. Deng et al. [14] reported a LOD score of about 1 on chromosome 18 at 75 cM. When comparing the results from these genome scans, differences between the studies have to be considered. While all but one study investigated individuals of Caucasian origin (Thompson et al. [12] analyzed Pima Indians), differences in sampling, sample size, pedigree structure, and marker sets were more pronounced. Therefore, a meta-analysis of all seven genome scans for the phenotype stature is desirable to quantify exactly the statistical evidence for linkage in these regions.

The power of linkage analysis was substantially reduced since for many founders no DNA was available. Nevertheless, this population-based and unselected sample has been a good example for the successful identification of linked regions. In particular, we consider the regions on chromosomes 6 and 16 for BMI and the regions on chromosomes 6, 9, and 12 for stature interesting for fine mapping and candidate gene studies. Our results indicate that for moderately to highly heritable traits the analysis of phenotypically well characterized but unselected and rather large samples of extended pedigrees is promising. Other such large epidemiological cohort studies, where many covariables are carefully collected, can be valuable and efficient tools in studying the genes and interactions between genes and environmental factors in common complex diseases.

## Figures and Tables

**Table 1:** Multi-point LOD scores > 1 for BMI with different methods.

			**LOD Scores**		
			
**Chromosome**	**Marker**	**Peak Position (cM)**	**SOLAR**	**MERLIN-VC**	**MERLIN-REGRESS**
1	D1S1728	120	0.57	1.33	1.31
1	D1S2141	258–259	1.52	1.56	1.35
2	D2S2976	11–12	1.68	1.52	1.44
4	D4S2361	106–109	1.18	1.08	0.91
5	D5S1719	88–99	1.48	1.29	1.13
6	D6S1009	150–154	1.90	2.70	2.13
8	D8S1106	29–33	1.25	1.45	1.07
9	D9S1120	97–110	1.29	1.14	1.00
16	D16S3253	64–79	3.21	2.81	2.47

**Table 2:** Multi-point LOD scores > 1 for adult height with different methods.

			**LOD Scores**		
			
**Chromosome**	**Marker**	**Peak Position (cM)**	**SOLAR**	**MERLIN-VC**	**MERLIN-REGRESS**
6	D6S2434	23–28	1.24	1.34	1.36
6	GATA184A08	159–162	1.19	1.06	1.14
6	D6S503	200–201	1.83	1.67	2.45
9	D9S319	60–61	1.22	1.15	1.56
12	D12S398	70–77	0.48	1.33	1.70
14	D14S742	5–11	1.27	0.80	1.02
14	D14S1426	137	1.35	1.49	1.58
18	D18S1364	111	1.54	1.44	1.73
22	D22S345	19–21	1.07	1.16	1.28

**Table 3:** Multi-point LOD scores for adult height in different scans.

**Chromosome**	**Region (cM)**	**Our study**	**Xu et al.**	**Hirschhorn et al.**	**Perola et al.**
6p	0–30	1.36	<1	1.08	1.4
6q	155–200	2.45	3.06	3.85	0
9	42–66	1.53	2.09	2.01	0.15
12	56–80	1.55	1.86	3.35	0.82
14	11–47	1.02	<0.8	<1	1.67
18	72–116	1.73	<0.5	1.77	1.71
22	0–27	1.28	<0.8	1.95	0.54

Upper limits for the LOD scores < 1 from Xu et al. [10] and Hirschhorn et al. [9] were estimated from their figures.
